# Telomere Length in Relation to Immunological Parameters in Patients with Renal Cell Carcinoma

**DOI:** 10.1371/journal.pone.0055543

**Published:** 2013-02-01

**Authors:** Ulrika Svenson, Elisabeth Grönlund, Ingegerd Söderström, Raviprakash T. Sitaram, Börje Ljungberg, Göran Roos

**Affiliations:** 1 Department of Medical Biosciences, Pathology, Umeå University, Umeå, Sweden; 2 Hematopathology Laboratory, University Hospital, Umeå, Sweden; 3 Department of Public Health and Clinical Medicine, Medicine, Umeå University, Umeå, Sweden; 4 Department of Surgical and Perioperative Sciences, Urology and Andrology, Umeå University, Umeå, Sweden; Health Canada, Canada

## Abstract

Over the last decade, telomere length (TL) has gained attention as a potential biomarker in cancer disease. We previously reported that long blood TL was associated with a poorer outcome in patients with breast cancer and renal cell carcinoma. Based on these findings, we hypothesized that certain immunological components may have an impact on TL dynamics in cancer patients. One aim of the present study was to investigate a possible association between serum cytokines and TL of peripheral blood cells, tumors and corresponding kidney cortex, in patients with clear cell renal cell carcinoma. For this purpose, a multiplex cytokine assay was used. Correlation analysis revealed significant positive correlations between tumor TL and peripheral levels of three cytokines (IL-7, IL-8 and IL-10). In a parallel patient group with various kidney tumors, TL was investigated in whole blood and in immune cell subsets in relation to peripheral levels of regulatory T cells (Tregs). A significant positive association was found between whole blood TL and Treg levels. However, the strongest correlation was found between Tregs and TL of the T lymphocyte fraction. Thus, patients with higher Treg levels displayed longer T cell telomeres, which might reflect a suppressed immune system with fewer cell divisions and hence less telomere shortening. These results are in line with our earlier observation that long blood TL is an unfavorable prognostic factor for cancer-specific survival. In summary, we here show that immunological components are associated with TL in patients with renal cell carcinoma, providing further insight into the field of telomere biology in cancer.

## Introduction

Telomeres, which consist of repetitive TTAGGG sequences and specific proteins, are located at the ends of eukaryotic chromosomes, forming a capping structure that prevents chromosomal damage and degradation [1]. Telomeric repeats are normally lost during each cell division, unless the cell has mechanisms for telomere maintenance, e.g. through activation of the enzyme telomerase [2]. Telomerase, which acts by adding TTAGGG repeats to the telomeres, is inactive in most normal cells except for in e.g. germ cells, stem cells and activated lymphocytes, but the majority of cancer cells exhibit telomerase activity, thereby achieving unlimited replicative potential [3]. Telomere length homeostasis is a complex process affected by both intrinsic and extrinsic factors, such as heredity, epigenetics and environmental factors, including inflammation and stress [4].

Renal cell carcinoma (RCC) accounts for ∼3% of all adult cancers worldwide and nearly one-third of the patients have metastasis at the time of diagnosis [5]. A broad variety of diagnostic and prognostic molecular markers for RCC have been described in the literature, such as various RCC-associated tissue factors and molecular markers in blood/serum and urine [6], but ideal biomarkers for clinical practice are still lacking. In recent years, there has been a growing interest in investigating telomere length (TL) as a possible biomarker in malignancy (as previously reviewed in [7]–[9]). We recently reported that blood cell TL, measured by qPCR as relative TL (RTL), was associated with survival in newly diagnosed patients with breast cancer [10] and clear cell RCC (ccRCC) [11]. Patients with long blood RTL had a significantly worse outcome compared to those with shorter blood RTL. In our ccRCC study [11], neither RTL in tumor tissue nor RTL in corresponding kidney cortex could predict outcome per se, but a non-significant trend towards a worse outcome was observed in patients with a high tumor-to-nontumor (T/N) RTL ratio. In that paper, we speculated that our observed association between long blood telomeres and a worse outcome could reflect a suppressed immune response in a subset of cancer patients, leading to less telomere attrition due to fewer cell divisions [11]. In addition, various telomerase-stimulating factors might have been present in increased levels in some patients. For example, a number of cytokines have been shown to upregulate the activity of telomerase, including interleukin (IL)-2, IL-4, IL-6, IL-7, IL-10, and IL-13 [12]–[16].

The role of the immune system in cancer disease is complex. It is well known that tumor cells can develop mechanisms to escape the immune system and several suppressive mechanisms have been described in RCC [17]. For example, an increased frequency of regulatory T cells (Tregs) has been reported in RCC patients [18], as well as in other malignancies [19]–[23]. The role of cytokines in malignant disorders is dual. On one hand, cytokines can suppress the formation of cancer cells by controlling inflammation and immunity. On the other hand, cancer cells can exploit cytokines to favor tumor development and progression [24].

One of the aims of the present study was to investigate a potential relationship between serum cytokine levels and TL of peripheral blood, tumor and non-malignant kidney cortex tissue. We therefore used a multiplex bead array assay to analyze 17 cytokines in sera of the same population of ccRCC patients as previously described [11]. Telomerase activity (TA), measured in tumor tissue of a subset of the patients, was included as an additional parameter. Moreover, to further explore a potential association between immunological mechanisms and blood TL, a parallel patient group with newly diagnosed RCC tumors was investigated with regard to TL in whole blood and blood cell subsets in relation to peripheral levels of CD4+CD25^high^CD127^low/-^ Treg cells.

To our knowledge, this is the first study to investigate peripheral levels of cytokines and Tregs in relation to TL in patients with RCC.

## Materials and Methods

### Ethics statement

The study was approved by the regional ethical review board in Umeå (Dnr 07-071M and 07-072M), and each patient participated after providing informed and signed consent.

### Patients

The study material included two populations:

**“Study 1”** originally included 105 patients diagnosed with ccRCC between the years of 2001 and 2007. This study population has previously been described in [11]. Serum samples were available for 102 patients (60 men and 42 women, median age 65 years) and these samples were included in the cytokine measurements. In addition, CHAPS extracts from 35 patients were available for telomerase activity analysis.

**“Study 2”** consisted of 51 patients (30 men and 21 women, median age 68 years) diagnosed with RCC between the years of 2008 and 2010. The tumor subtypes included: clear cell RCC (n = 32), papillary RCC (n = 9), chromophobe RCC (n = 2) and oncocytoma (n = 8).

In brief, patients were nephrectomized at the Department of Urology, Umeå University Hospital, Umeå, Sweden, and follow-up was performed with regular clinical and radiological examinations. RCC subtype classification was performed according to the Heidelberg consensus conference [25]. Upon extirpation, tissue samples were snap-frozen in liquid nitrogen and stored in −80°C until analysis. Blood samples were collected prior to any systemic therapy.

### Multiplex cytokine analysis (Study 1)

Seventeen cytokines were analyzed in serum samples using the Bio-Plex™ Suspension Array System from Bio-Rad (Hercules, CA, USA). The 17-plex panel included IL-1β, IL-2, IL-4, IL-5, IL-6, IL-7, IL-8, IL-10, IL-12, IL-13, IL-17, G-CSF, GM-CSF, IFN-γ, MCP-1, MIP-1β and TNF-α. The assay was performed according to the instruction manual, except that each serum sample was diluted 1∶3 in sample diluent, as described in [26]. In addition, an internal control was included in all runs, consisting of four pooled patient serum samples. All samples, including the high PMT standards, were assayed in duplicate and analyzed with a Luminex 200 Labmap system. Data was evaluated using the Bio-Plex Manager software version 4.1.1 (Bio-Rad).

### Telomerase activity measurement (Study 1)

Telomerase activity was measured in CHAPS extracts (250 ng) from ccRCC tumor tissue (n = 35), using a quantitative telomerase detection kit (QTD kit, Allied Biotech Inc). QTD real-time PCR assays were performed on the ABI Prism 7900 HT sequence detection system (Applied Biosystems) according to the manufacturer's protocol. The kit also includes a TSR control template, providing standard curve and positive control. Telomerase activity was evaluated using the ABI Prism SDS Software v. 2.4 (Applied Biosystems).

### Cell separation and DNA extraction (Study 2)

Peripheral blood leukocytes were separated using Dynabead-coupled antibodies (Dynal Biotech Dynabeads, Norway) against surface markers CD3 (T cells, Cat. No. 111.51) and CD19 (B cells, Cat. No. 111.43). Separation was performed according to the manufacturer's protocol. The resulting cell fractions consisted of T cells, B cells and the remaining myeloid cells (“M fraction”, CD45+). DNA extraction was performed on whole blood and on the three cell fractions using the BioRobot M48 Workstation with MagAttract technology, as described elsewhere (Qiagen, Germany). A few samples were excluded due to poor DNA yield, whereas the remaining DNA samples were included in the telomere length analysis described below (whole blood: n = 50, B fraction: n = 44, T fraction: n = 50, M fraction: n = 47).

### Flow cytometry for immunophenotype analysis (Study 2)

Blood samples were analyzed by flow cytometry within 24 (–48) hours from blood sampling. Until preparation and analysis, all samples were kept in EDTA-K3 anticoagulation vacuum tubes. The antibodies used were fluorochrome-labeled mouse anti-human monoclonal antibodies targeted against: CD8-FITC (BDBiosciences), CD127-PE (BDPharmingen), CD4-PerCp (BDBiosciences), CD19-PECy7 (Beckman Coulter), CD25-APC (BDBiosciences), CD3-APCAlexa 750 (Beckman Coulter), CD16-Pacific Blue (BDBiosciences) and CD45-AmCyan (BDBiosciences). In brief, blood samples (50 ul) were transferred and incubated with antibodies at room temperature (15 min in the dark), followed by lysing with ammonium chloride. After two rounds of centrifugation (5 min at 400 g) and washing in PBS, cells were resuspended in 300 ul of PBS before tested by flow cytometry. All detections were performed on a FACSCantoII flow cytometer and analyzed by the FACSDiva software (BD Biosciences). Lymphoid cells were identified according to their strong CD45 expression and low side scatter. Tregs were identified as the CD4+CD25highCD127low/- cell subset by subsequent gating,

### Relative telomere length measurements

Relative telomere length of peripheral blood, tumor tissue and kidney cortex samples had previously been measured by qPCR on the patients of Study 1, as described in [11]. The same method was used when analyzing telomere length on DNA samples from patients of Study 2. For a more detailed description, see refs [11] and [27]. Briefly, the ratio of telomere repeat copy number to single-copy gene number (T/S ratio) was determined using the comparative Ct method. Sample T/S ratios were then divided with the T/S ratio of a reference DNA included in each plate, generating relative telomere length values (RTL). Each sample was loaded in triplicate and all PCR-plates included a standard curve for PCR efficiency calculations. In our laboratory, the inter-assay coefficient of variation (CV) for this method ranges between 4–8% [10], [11], [28].

### Statistical analysis

All statistical calculations were performed using PASW Statistics 18.0 (SPSS Statistics).

#### Study 1

In the cytokine analysis, three patients were found to be extreme high outliers and were removed from further analysis. The remaining 99 patients were included in the statistical calculations. Because of a skewed distribution of cytokine concentrations, Spearman's rank correlation test was used to calculate the correlation between telomere length, telomerase activity and cytokine levels. Between-group differences were investigated by analysis of covariance (ANCOVA) with age as a covariate, using ln-transformed RTL and TA values.

#### Study 2

Correlations were investigated by Pearson's correlation coefficient, using ln-transformed RTL values. Partial correlation with age-adjustment was performed when appropriate. Mean RTL values were compared by two-tailed unpaired and paired *t* tests.

## Results

### Relationship between cytokines, telomere length and telomerase activity

**Figure 1:**
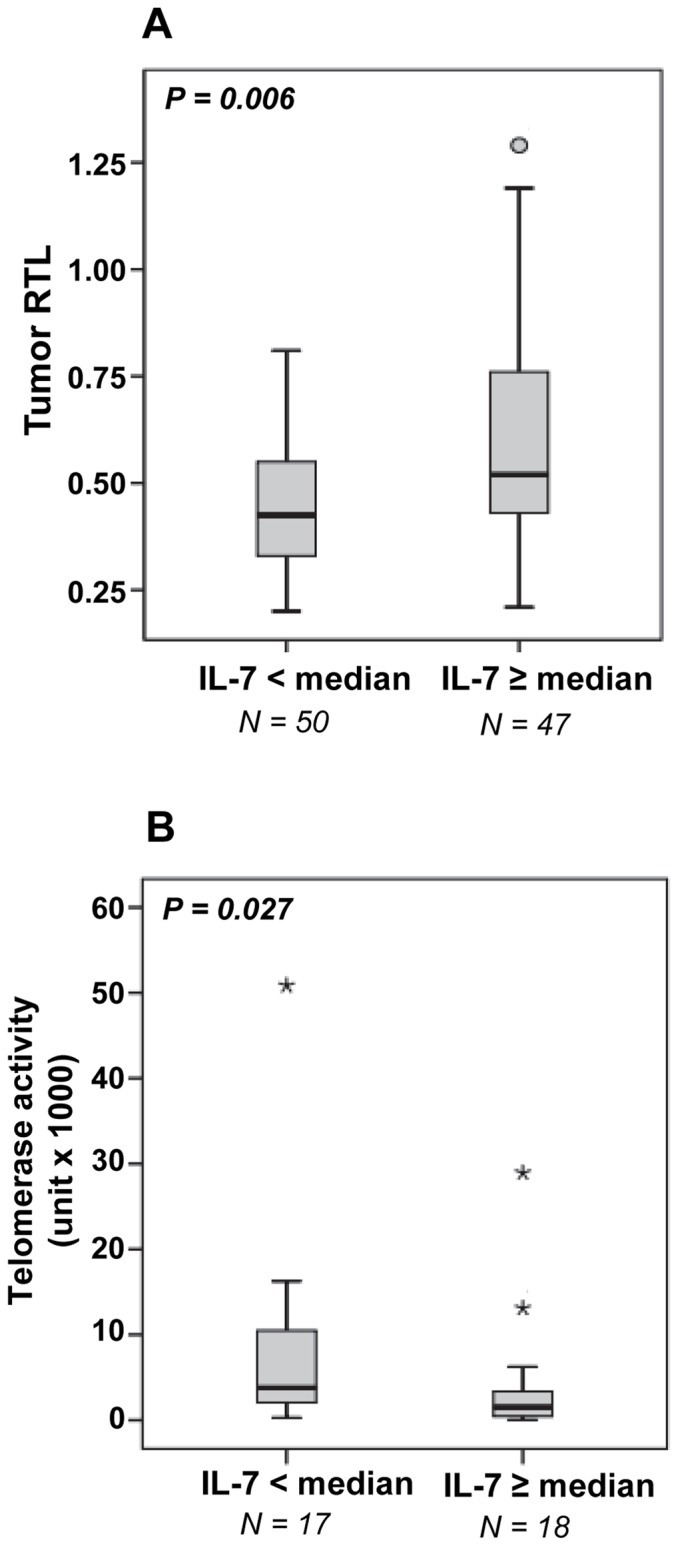
Tumor telomere length and telomerase activity in relation to IL-7. Comparison of (A) tumor RTL and (B) tumor TA between patients with IL-7 levels below vs. above median (79 pg/ml). Differences between the groups were compared by ANCOVA with age-adjustment.

Nine cytokines (IL-1β, IL-2, IL-4, IL-12, IL-13, IL-17, GM-CSF, IFN-γ and TNF-α) were detectable above the lowest point on the standard curve in less than 10% of the ccRCC patients of Study 1. These cytokines were therefore excluded from further statistical calculations. The remaining cytokines were: IL-5, IL-6, IL-7, IL-8, IL-10, G-CSF, MCP-1 and MIP-1β. Descriptive statistics for these cytokines are found in Table 1. Only IL-7 showed a significant association with age (r = −0.216, P = 0.032) (data not shown). A summary of correlation investigations between cytokine levels and RTL values in blood, kidney cortex tissue and tumor tissue is presented in Table 2. In addition, the T/N RTL ratio and tumor TA were included as parameters. Tumor RTL and T/N RTL ratio correlated positively with three of the cytokines (IL-7, IL-8 and IL-10), whereas TA correlated inversely with IL-7 and IL-8. The results are further exemplified in Figure 1, showing the difference in tumor RTL (Fig. 1A) and tumor TA (Fig. 1B) between patients with low vs. high levels of IL-7 (cut-off = the median value of 79 pg/ml). Neither blood RTL nor RTL in kidney cortex tissue correlated to any of the cytokines. A significant inverse correlation was found between tumor TA and T/N RTL ratio (r = −0.413, P = 0.023), whereas no significant correlations were observed between tumor TA and RTL in blood (r = 0.007, P = 0.966), kidney cortex (r = 0.093, P = 0.614), or tumor tissue (r = −0.307, P = 0.083) (data not shown). It should, however, be noted that TA data was available for only 35 patients.

**Table 1:** Serum cytokine levels (pg/ml) in 99 patients with ccRCC (Study 1).

Analytes	Mean	Median	Minimum	Maximum
IL-5	2.9	2.1	2.1*	15.1
IL-6	101.9	33.8	2.4*	751.9
IL-7	91.6	78.8	22.3	250.8
IL-8	77.4	58.5	14.9	303.0
IL-10	3.57	1.68	1.68*	30.1
G-CSF	30.7	18.7	1.58*	289.8
MCP-1	261.7	237.8	2.15*	965.0
MIP-1β	739.1	694.8	209.7	2119.4

***NOTE:*** *IL-5 = interleukin-5; G-CSF = granulocyte colony-stimulating factor; MCP-1 = monocyte chemoattractant protein 1; MIP-1β = macrophage inflammatory protein 1-beta.*

* *Minimum value = lowest point on the standard curve.*

**Table 2:** Correlation estimates (r) between serum cytokine levels versus RTL in blood, tumor and kidney cortex tissues, T/N RTL ratio and tumor TA, in patients with ccRCC.

			RTL blood	RTL kidney cortex	RTL tumor	T/N RTL ratio	Tumor TA
			(*N = 99*)	(*N = 87*)	(*N = 97*)	(*N = 85*)	(*N = 35*)
Spearman's rho	**IL-5**	r	.091	.116	.058	−.011	0.055
		*P*	.370	.286	.573	.924	0.755
	**IL-6**	r	.009	−.075	.086	.187	−0.166
		*P*	.929	.490	.403	.087	0.341
	**IL-7**	r	.112	.123	.306	.333	−0.458
		*P*	.268	.258	.002*	.002*	0.006*
	**IL-8**	r	−.075	.042	.240	.234	−0.426
		*P*	.458	.702	.018*	.031*	0.011*
	**IL-10**	r	.127	.030	.341	.374	−0.149
		*P*	.211	.782	.001*	.000*	0.393
	**G-CSF**	r	−.068	−.125	.009	.066	0.030
		*P*	.501	.247	.928	.548	0.866
	**MCP-1**	r	−.009	.152	.145	.014	0.003
		*P*	.931	.160	.155	.898	0.985
	**MIP-1β**	r	−.082	.021	.037	.081	−0.101
		*P*	.419	.844	.717	.463	0.565

Significant *P* values (≤0.05) are marked with an asterisk.

### Telomere length of blood cell subpopulations and associations with Treg levels

The Study 2 patient group (n = 51) comprised patients with different RCC tumor types, the majority diagnosed with ccRCC (n = 32). The other tumor types included papillary RCC (n = 9), chromophobe RCC (n = 2) and oncocytoma (n = 8). Because of the small sample size of most subgroups, statistical calculations were performed on the group as a whole in order to gain more statistical power. However, restricting the analyses to the largest subgroup (ccRCC) did not change the results presented below (data not shown).

**Figure 2:**
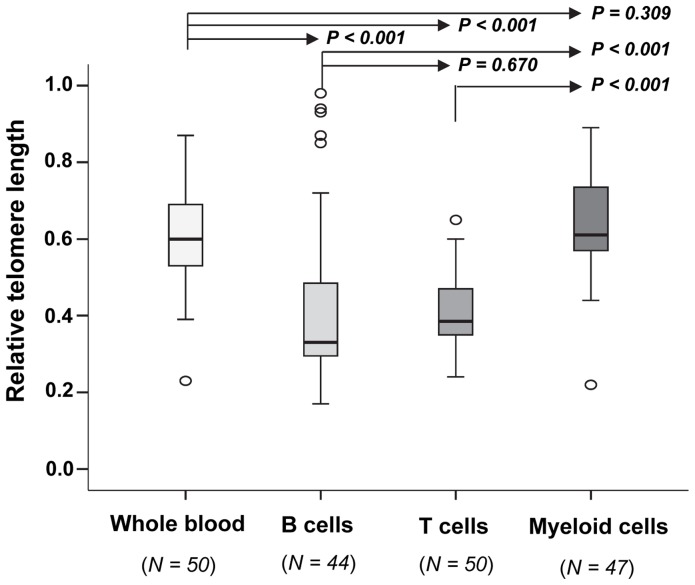
Telomere length in whole blood and in blood cell subpopulations illustrated by box-plots. Mean values were compared by independent samples *t* test.

As Figure 2 demonstrates, significantly longer telomere length (P<0.001) was found in the myeloid cell fraction and in whole blood, as compared to the B and T cell fractions. No significant differences in mean RTL were found between B and T cells (P = 0.670), or between the M fraction and whole blood (P = 0.309). Paired samples test showed that the same pattern could be seen also at the individual level [i.e. RTL of whole blood and myeloid cells>RTL of B and T cells (P<0.001)]. Additional information is given in Table 3, showing RTL data and percentage of Tregs (expressed as percentage of total blood leukocytes) for this patient group.

**Table 3:** Descriptive data regarding relative telomere length and % Tregs estimated in peripheral blood from 51 patients with RCC (Study 2).

		Relative telomere length (RTL)				% Tregs (of total)
		Whole blood	B-cells	T-cells	M-cells	
**Mean (± SD)**		0.61 (0.12)	0.42 (0.21)	0.40 (0.10)	0.64 (0.13)	1.09 (0.54)
**Minimum**		0.23	0.17	0.24	0.22	0.10
**Maximum**		0.87	0.98	0.65	0.89	2.60
***N***	*Valid*	*50*	*44*	*50*	*47*	*49*
	*Missing*	*1*	*7*	*1*	*4*	*2*

Age was significantly and inversely correlated with T cell RTL (r = −0.394, P = 0.005), but not with RTL in whole blood (r = −0.196, P = 0.172), B cells (r = 0.129, P = 0.405) or myeloid cells (r = −0.037, P = 0.805) (data not shown).

**Figure 3:**
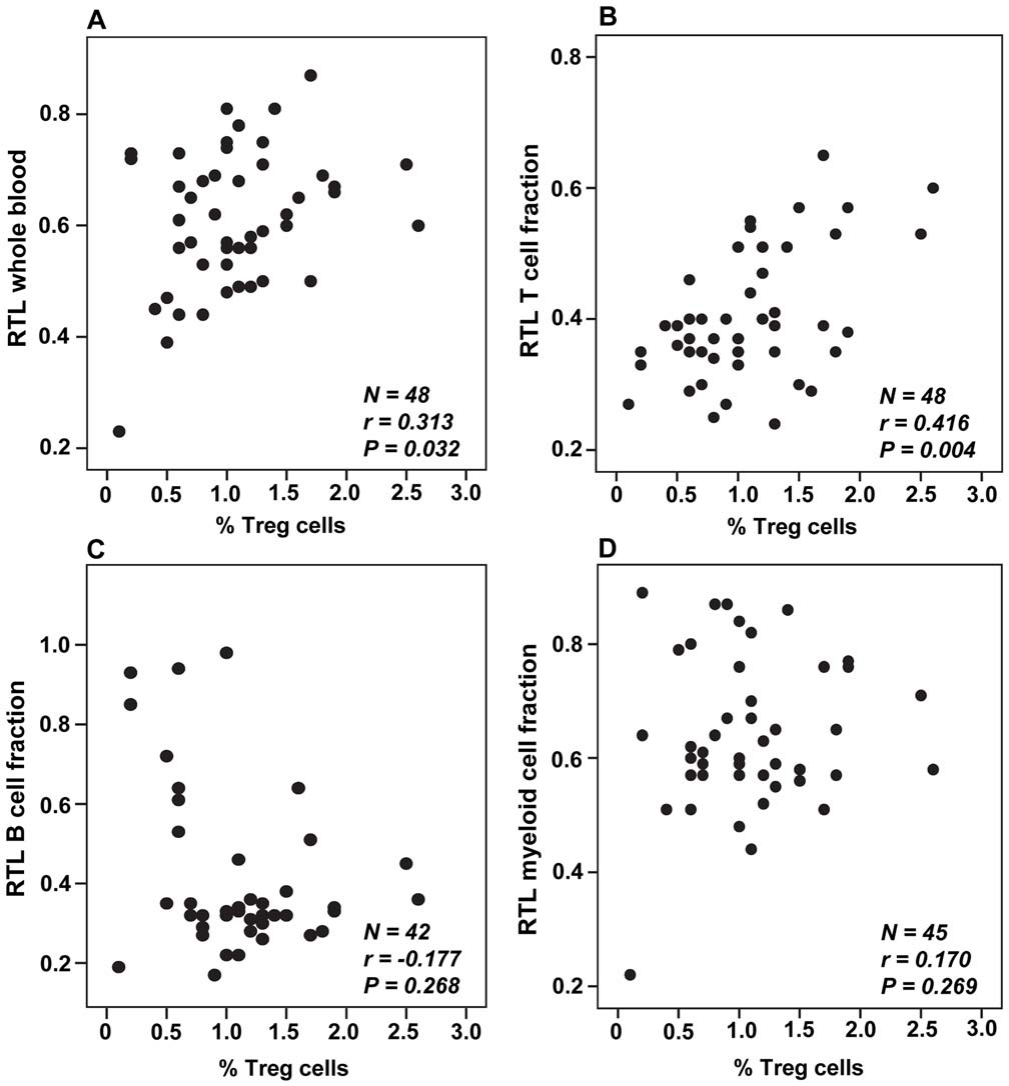
Correlations between telomere length and Treg levels. T-regs, expressed as the percentage of total blood leukocytes, were investigated in relation to (A) whole blood RTL, (B) B cell RTL, (C) T cell RTL and (D) M cell RTL, using Pearson's partial correlation with age-adjustment.

**Figure 4:**
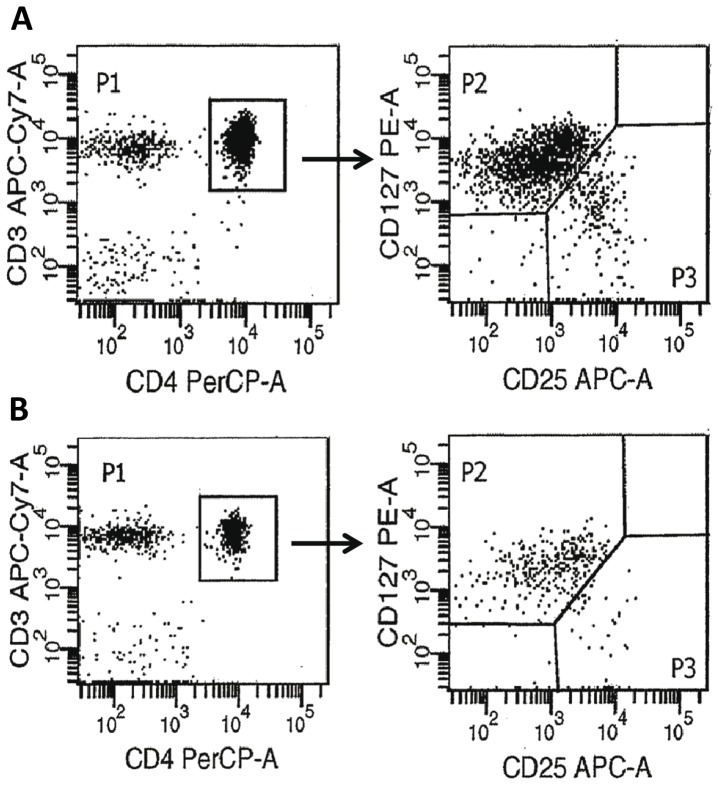
Flow cytometric plots for Treg evaluation. Gating on CD3+/CD4+ cells (left panel) was followed by subsequent gating according to the CD25 and CD127 expression (right panel). Tregs were defined as the CD4^+^CD25^high^CD127^low/-^ -cells (P3). (A) A patient with high Tregs/long T cell telomere length. % Tregs of total PBL: 1.7; % Tregs of CD4+ cells: 7.7; T cell telomere length: 0.65. (B) A patient with low Tregs/short T cell telomere length. % Tregs of total PBL: 0.2; % Tregs of CD4+ cells: 4.8; T cell telomere length: 0.33.

The relationship between RTL and Tregs is illustrated in Figure 3. Since a close to significant correlation was found between age and Treg levels (r = −0.267; P = 0.063), age-adjusted correlation calculations were performed. A significant positive correlation was found between Treg levels and whole blood RTL (r = 0.313, adjusted P = 0.032). An even stronger positive correlation was observed between Tregs and RTL of the T cell fraction (r = 0.416, adjusted P = 0.004), i.e. patients with a higher percentage of Tregs had longer T cell telomeres. In contrast, neither B cell RTL (r = −0.177, adjusted P = 0.268), nor RTL of the M fraction (r = 0.170, adjusted P = 0.269), correlated with Treg levels. Examples of flow cytometric plots for Treg evaluation can be viewed in figure 4, showing a patient with high Treg levels and long T cell RTL(A) and a patient with low Tregs and short T cell RTL (B).

## Discussion

In the present study, telomere length was investigated in relation to immunological parameters using two separate groups of patients diagnosed with RCC. One aim was to explore a potential relationship between serum cytokine levels and TL in peripheral blood and ccRCC tumor tissue. Interestingly, we found that IL-7, IL-8 and IL-10 all correlated positively with tumor RTL and T/N RTL ratio. These novel results indicate that a functional link may exist between these serum cytokines and ccRCC tumor TL. IL-7 is important in the regulation of T and B cell development and T cell homeostasis, and the cytokine is produced at various sites, including in bone marrow, keratinocytes and dendritic cells [29]. Elevated IL-7 levels have been associated with poor survival in for example ovarian cancer [30] and breast cancer patients [31]. IL-8 is a chemokine that exerts chemotactic effects on leukocytes. The cytokine is known to be involved in tumor growth and progression, e.g. through mitogenic and angiogenic effects [32]. IL-10 is expressed by several immune cells, including Tregs, and it can induce immune suppression in various ways, for example by inhibiting cytotoxic T lymphocyte activation [24]. IL-10 has been detected in increased levels in RCC patients [33]–[34], as well as in other malignancies [24].

As mentioned in the introduction, a number of cytokines have been shown to upregulate telomerase activity, IL-7 and IL-10 included. It can therefore be hypothesized that the accumulation of high concentrations of various telomerase-stimulating cytokines in the tumor microenvironment may have effects on the tumor TL. Adding to the complexity though, is the fact that tumor TA was inversely correlated to IL-7 and IL-8, as well as to T/N RTL ratio, in the present study. In addition, a trend towards an inverse correlation (P = 0.083) was found between tumor TA and tumor RTL. Thus, the observed positive correlation between tumor RTL and certain cytokines does not seem to be explained by increased TA. It is well documented that TA+ malignant tumors in general have shorter telomeres compared to corresponding normal TA- tissues, and for malignant cells there is no direct correlation between TA levels and TL. Hence, high TA levels do not necessarily correlate with long telomeres. In fact, a negative correlation between these parameters has been described for hematologic malignancies, e.g. chronic lymphocytic leukemia and multiple myeloma [35]–[36]. It should also be noted that TA was available for only 35 patients in the present study.

In our previous paper [11], we speculated that high serum cytokine levels might be associated with long telomeres in peripheral blood cells. In the present study, no such associations were observed. It might be assumed that that the blood cells (and their precursors) had been exposed to lower cytokine levels compared to the tumor cells. Also, since blood RTL reflects the mean RTL of a mixture of leukocyte subpopulations, it cannot be excluded that the RTL of e.g. lymphocytes alone would correlate to certain cytokines. Nevertheless, the cytokine-related results of the present study do not explain our previous findings that patients with long blood RTL had a poorer prognosis compared to patients with shorter blood RTL.

The other aim of the study was to investigate a possible relationship between RTL of blood cell subpopulations and peripheral Treg levels. We previously suggested that suppression of the immune response, e.g. through the action of Tregs, in a subset of cancer patients might lead to less telomere attrition in immune cells due to fewer cell divisions. Interestingly, we here found a positive correlation between whole blood RTL and Treg levels in peripheral blood. Perhaps the most striking finding was that the strongest correlation was found between the percentage of Tregs and T cell RTL. In a recent study by Liu et al. [37], long leukocyte RTL was associated with a worse survival in patients with hepatocellular carcinoma (HCC). Furthermore, and in line with our theory, they found that patients with long leukocyte RTL had a significantly increased percentage of Tregs. Their results in HCC patients are hence consistent with our results in patients with RCC. Tregs have been detected in increased levels in a large variety of cancers and they are believed to be key players in establishing tumor immune tolerance [38]. Mechanisms through which Tregs are believed to act include secretion of immunosuppressive mediators, such as IL-10 and TGF-beta, cytolytic activity and metabolic disruption of effector T cells [38]. In addition, Tregs have been found to contribute to tumor angiogenesis [38], further enabling tumor progression. The significant positive correlation between Treg amount and T cell RTL in the present study is plausible since T cells are important targets for Treg-mediated suppression. For this reason, it may not be surprising that no significant relationship was found between Tregs and RTL of the B and M cell fractions.

Regarding RTL of blood cell subpopulations, we found that B and T lymphocytes had significantly shorter mean RTL compared to the myeloid cell fraction, in which granulocytes are dominant. In agreement, previous and recent studies have reported that granulocytes have longer telomeres compared to lymphocytes in adults, and that lymphocytes exhibit a faster age-dependent decline in TL compared to granulocytes [39]–[41]. Of interest is also the fact that there is a shift from naïve T cells towards memory T cells with age, and memory T cells display shorter telomeres than naïve T cells [42]–[43]. Although our study population consisted of RCC patients, our results are in line with the previous studies mentioned above.

In conclusion, we here present novel data showing that peripheral levels of Tregs and certain cytokines are associated with TL in patients with renal cell tumors. Of special interest is our finding of a positive correlation between blood RTL and Treg cells, since it may provide a clue to our previous observations that long blood telomeres are associated with poorer cancer-specific survival. A limitation of the present study is the relatively small number of patients, limiting the statistic power of the study. Thus, in order to further establish the potential relationship between immunological components and TL, larger studies are warranted.
